# Sex-Differences in Discontinuation of Statin Treatment in Cancer Patients the Year before Death

**DOI:** 10.3390/ph14040368

**Published:** 2021-04-16

**Authors:** Gabriella Frisk, Helena Bergström, Maria Helde Frankling, Linda Björkhem-Bergman

**Affiliations:** 1 Division of Clinical Geriatrics, Department of Neurobiology, Care Sciences and Society (NVS), Karolinska Institutet, Blickagången 16, Neo Floor 7, SE-141 83 Huddinge, Sweden; gabriella.frisk@ki.se (G.F.); helena.bergstrom.1@ki.se (H.B.); maria.helde.frankling@ki.se (M.H.F.); 2 ASIH Stockholm Södra, Palliative Home Care and Hospice Ward, Bergtallsvägen 12, SE-125 59 Älvsjö, Sweden; 3 Stockholms Sjukhem, Palliative Medicine, Mariebergsgatan 22, SE-112 19 Stockholm, Sweden

**Keywords:** statins, palliative care, sex-differences, cancer care, deprescribing

## Abstract

Statin treatment is often terminated in patients with advanced cancer but guidelines for statin discontinuation are still lacking. The aim of this study was to investigate sex-differences in time-points of statin discontinuation in patients with advanced cancer. Medical records from 1535 deceased patients enrolled at a Palliative Home Care Unit were reviewed. A total of 149 patients (42 women and 107 men) who were diagnosed with cancer, and were treated with statins one year before death, were identified. Statin treatment was terminated earlier in women than in men, 3.0 months prior to death (IQR 0.88–7.25) as compared to 1.5 months (IQR 0.5–4.0) (*p* < 0.05), respectively. In a longitudinal analysis there was a significant difference between men and women still on statin treatment at all studied time-points, 9, 6, and 3 months before death (*p* < 0.05), where women terminated statin treatment earlier in the disease trajectory. Baseline demographics were similar between the sexes except that more men than women had a history of previous cardiovascular events (*p* < 0.01). However, neither the indication for statin treatment, i.e., primary prevention versus secondary prevention, nor age could explain the sex-difference in statin discontinuation. There was no difference in cardiovascular events or mortality between men and women after statin discontinuation.

## 1. Introduction

Preventive medications to lower cholesterol and blood pressure can often be discontinued in patients with advanced cancer since they are considered to do more harm than good. However, studies show that such medications are generally discontinued very late in the disease trajectory [1,2,3,4]. This may be due to patient perception of deprescribing, to the physician’s opinion on the effects of statins in the elderly or to physician fear of causing harm to the patient [5,6,7]. 

There are so far no guidelines based on randomized controlled trials to support physician and patients in the decision to terminate statins in palliative cancer patients [8,9]. However, in recent European expert consensus on inappropriate drug prescribing for people aged >75 years, lipid lowering agents were deemed questionable [10]. The consensus was based on criteria from the Screening Tool of Older Persons’ Potentially Inappropriate Prescriptions (STOPP) and involved patients with limited life expectance of ≤3 months [11]. 

In a randomized, unblinded, clinical trial on statin discontinuation in 381 palliative care patients performed by Kutner et al., there was no statistically significant difference in time to death between patients who continued statins and those who terminated, 23.8% vs. 20.3 (*p* = 0.39) [12]. Interestingly, overall Quality of life (QoL) was significantly higher amongst patients discontinuing statins.

Preclinical studies have suggested that loss of anti-inflammatory and immunomodulatory effects of statins, so called pleiotropic effects, may increase the risk of CVD events also short term [9,13,14,15]. This theory is supported by a limited number of retrospective observational studies in patients where statin discontinuation increased the risk of worse outcomes [15,16]. Moreover, in a randomized controlled trial (RCT) of statin discontinuation (*n* = 43) versus continuation (*n* = 46) after acute ischemic stroke, discontinuation was associated with a significantly increased risk of death [17]. However, no increase in adverse outcomes have been observed when discontinuing statins in patients with stable coronary heart disease [15]. 

Interestingly, few studies have examined sex-differences in discontinuation of statin treatment. In a small pilot-study (*n* = 52) from a palliative unit in Stockholm, Sweden, we previously reported that statin treatment was terminated significantly earlier in women than in men [18]. In this study, there were no differences in age, socioeconomic factors or indication for statin treatment that could explain the difference in time-point for statin termination. However, this was a small cohort and performed as a post hoc analysis of a previously performed clinical trial. 

With this background, we conducted a larger, observational study in patients with advanced cancer, admitted to our Palliative and Supportive Home Care Unit. The aim was to confirm or refute our previous results on possible sex-differences in statin discontinuation in cancer patients. 

## 2. Results

### 2.1. Study Cohort 

Medical records from 1535 patients were reviewed and 149 patients fulfilled the inclusion criteria. Patients diagnosed with cancer one year before death, treated with statin and admitted to Palliative and Supportive Home Care were eligible. Of the 149 patients, 42 were women and 107 were men. Baseline characteristics, including age, type of statin, indication for statin use, presence of previous cardiovascular events and type of cancer are presented in Table 1. Simvastatin was the most common prescribed statin in both women and men and there were no statistically significant difference in type of statins or doses of statins prescribed in women and men. In addition, there were no differences in type or doses of statins used regarding type of cancer. More men than women had a history of previous myocardial infarction (*p* < 0.05), and the indication for statin treatment was more often secondary prevention among men than among women (*p* < 0.01). Urological cancer was significantly more common in men than in women (Table 1).

### 2.2. Statin Discontinuation in Women and Men

**Figure 1:**
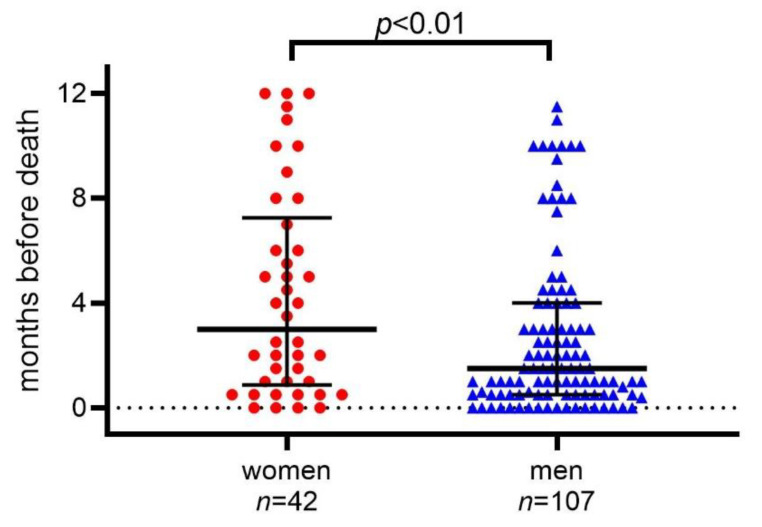
Time-point for statin termination during the year before death in 149 cancer patients admitted to Palliative Home Care. Lines show median and interquartile range.

Statin use was terminated earlier in women than in men. The median time for termination was 3.0 months before death in women compared to 1.5 months in men (*p* = 0.01) (Figure 1). 

**Figure 2:**
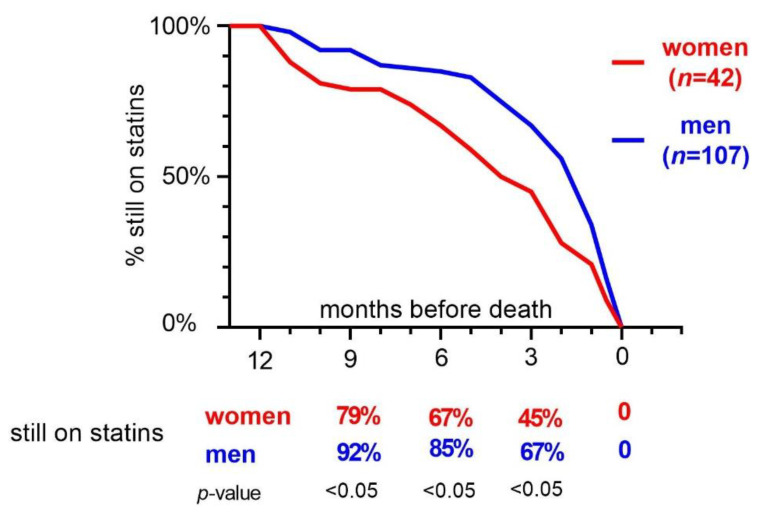
Time-graph when statin was terminated in men (*n* = 107) and women (*n* = 42) in relation to death during the last year in life. All patients had a cancer diagnosis more than one year before death.

The longitudinal analysis presented in Figure 2 shows a significant difference between men and women still on statin treatment at all studied time-points 3, 6, and 9 months before death (*p* < 0.05), whereas men discontinued their statin treatment later during the cancer disease. A subgroup analysis of women showed no statistically significant difference in time-point for statin discontinuation, regardless of whether the indication was primary or secondary prevention (Table 2).

In a similar manner the indication for statin use did not affect the time-point for discontinuation in men (*p* = 0.43). When comparing time-points for statin discontinuation between men and women with secondary prevention, the median time-point for statin termination was 1 month before death in men compared to 2.5 months in women. This difference did however not reach statistical significance (*p* = 0.06). There was generally a lack of data as to why statin treatment was discontinued, and this parameter had to be excluded from the analysis. There were 71 different physicians deprescribing statin treatment, including palliative care physicians at the Medical Home Care Unit, oncologists, primary care physicians and cardiologists. In 51% of the cases, statin treatment was deprescribed by a physician at the Palliative Care Unit. 

### 2.3. Indication for Statin Treatment in Relation to Statin Discontinuation

We performed an analysis to assess whether primary or secondary prevention affected the time-point for statin discontinuation in relation to death in the whole cohort. There was no significant difference between proportions of patients receiving primary or secondary prevention still treated with statins at time of death at any time-point or period prior to death (time of to death, ≤0.5, 0.5–1 month, 1–3, 3–6, 6–9, or more than 9 months). A multivariate regression analysis to investigate whether age, sex and type of indication for statin use affected the timepoint for discontinuation could not be performed, as data were heavily skewed due to many patients continuing their treatment until time if death. In addition, the number of observations was low. Thus, only univariate analyses were performed.

### 2.4. Cardiovascular Events and Causes of Deaths

Two men in the study cohort died due to acute myocardial infarction (AMI). One male patient on secondary prevention terminated his statin treatment 10 months before death, while the other male patient with AMI remained on statin treatment until the very last day of life. Three men died due to chronic heart failure, three due to renal failure unrelated to statin treatment, one of pneumonia and one of sepsis. All other men (*n* = 97) died of their cancer diseases according to the death certificates. One woman died of stroke. Her primary prevention treatment with statin was also terminated 10 months prior to death. Another female patient died of renal failure unrelated to statin treatment and all other women (*n* = 40) died of their cancer diseases according to the death certificates (Table 2).

## 3. Discussion

In the present study, we show that statin treatment was discontinued earlier in women than in men suffering from advanced cancer disease. The difference in time point for discontinuation of statins in relation to time of death could not be explained by age or indication for statin treatment, i.e., primary or secondary prevention. 

The results from this study, is in concordance with our previous, smaller study, which showed that statins were discontinued earlier in women than in men [18]. However, in that study patients with advanced cancer two years before death were included and they followed for the two last years in life compared to the last year in life in this study. In the previous study, statins were discontinued already 10 months before death in women compared to 3 months in this study.

The observation that statins were deprescribed on average 1.5 to 3 months before death in men and women respectively, are in line with a larger, population-based Swedish study, in which 52% of cancer patients were on statin treatment 90 days before death [2]. However, in that study no analysis of gender differences was made. Indeed, there is a lack of previous studies on sex-differences in statin deprescribing and in previous observational studies most participants have been males [19,20].

In the present study, there was no difference in cardiovascular events or cardiovascular mortality between men and women after statin discontinuation. This is also in line with earlier studies showing that statin discontinuation in palliative care patients do not affect mortality or cardiovascular events [12,18]. 

Interestingly, studies in patients with atherosclerotic cardiovascular disease show that women are less likely than men to be prescribed statins to prevent atherosclerotic cardiovascular disease [21,22]. Some researchers argue that there are no compelling data to support that statins are more harmful to women, while others argue that women are more likely to discontinue statins due to side effects [23,24,25].

In the study by Kutner et al., statin cessation was associated with improved QoL [12]. In our previous study there was no difference in self-assessed QoL between patients who continued and who had discontinued statin treatment [18]. The question whether statin treatment affects QoL or not is an important issue in the palliative care setting, but unfortunately, we had no data on this in the present, retrospective study. In addition, cost effectiveness is an issue to consider when continuing preventive medicine. This issue is currently studied in an ongoing study on statin cessation [26].

There is a risk that discontinuation of statin, may be perceived as the physician abandoning the patient [7]. It is therefore important to use patient-centered decision making and explain to the patient and next of kin that risk versus benefit for drug treatment have changed and that the treatment may now do more harm than good. At the same time, many patients experience statin discontinuation as it as a relief [7]. In fact, non-statin co-medication has been shown to decrease after statin deprescribing [12]. Finally, appetite and nutritional intake may decline at the end of life, making it difficult for the patient to ingest many tablets, and ultimately affecting the QoL.

During recent years, several studies have indicated that statin use may be beneficial in cancer disease [13,27,28]. This is also supported by several mechanistic studies in vitro and in vivo, suggesting different possible effects on inhibition of cancer growth and progression [28,29] as well as beneficial effects on oncological treatments [30]. However, there are today no general recommendations to continue statin treatment for cancer-preventive effects. In addition, possible beneficial effect of statin in cancer disease is probably of limited importance in the late palliative phase.

Like many other studies, our study has limitations. The study population is small (*n* = 149), and thus the number of events is low. Due to the retrospective design based on data from the medical records there was a lot of missing data. Especially, data on possible side effects due to statin treatment was missing, introducing an uncertainty to the reason for discontinuation of the treatment. In addition, data on fragility and performance status would have been of interest but were not possible to extract from the medical records. Finally, this is a single centre study which may introduce selection bias when it comes to patient socioeconomic background. 

In the future there is a need for prospective studies to confirm our result and to clarify how deprescribing affect the QoL in palliative patients and the risk of cardiovascular events. There is one ongoing randomized controlled trial on statin discontinuation in elderly >75 years [26]. However, in the previously mentioned randomized study performed by Kutner, more than 30% of the patients allocated to discontinuation restarted their treatment with statin [12]. This phenomenon may be difficult to address, and how to best design of deprescribing trials is a matter of discussion in the scientific community [9]. Thus, there is still a need for performing retrospective studies, especially in the palliative care setting. Finally, qualitative studies might be of use to further explore the reason for why physicians and patient, decide to terminate statins earlier in women than in men. 

There is no obvious explanation for why statins are discontinued earlier in women than in men with advanced cancer but we hypothesize that statins might be considered as doing more good than harm in men with cancer but the opposite is considered for women. An alternative hypothesis is that the remaining survival time might more often be overestimated in men than in women. This hypothesis is supported by two Swedish studies showing that men generally have poorer prognosis than women in several cancer forms [31,32].

The results presented here are in line with previous studies indicating that statins can be safely discontinued in patients with advanced cancer with limited life-time expectancy [11,12,18]. However, men and women are different populations and results from men might not necessarily be applicable on women. Anyhow, deprescribing statins might be considered earlier in the disease trajectory also in men.

## 4. Materials and Methods

### 4.1. Study Cohort and Study Site

Medical records from all enrolled patients at a Palliative and Supportive Home Care unit in Stockholm, Sweden (ASIH Stockholm Södra) between 2016 and 2018 were reviewed. This unit offers hospital-like palliative and supportive care to both oncological and non-oncological patients in their own homes. The unit also has an in-patient Hospice Ward with 16 beds. Most patients suffer from cancer, usually at an advanced stage. The median enrollment time is 3–4 months, but can vary from days to years depending on the need of supportive care. Most cancer patients stay enrolled at the unit until death. 

Patients enrolled at the unit who were on statin treatment and who were diagnosed with cancer one year prior to death were regarded as “statin users”. The time-point for deprescribing of statin therapy was identified and correlated to time of death. The patients were recruited consecutively and could have any type of cancer. Patients who had discontinued statin treatment before they were diagnosed with cancer were not included in the analysis, and neither were patients diagnosed with cancer less than one year before death.

### 4.2. Data Extraction

Medical records data were collected regarding age, sex, cancer diagnosis, statin type and dose, indication for statin treatment (primary or secondary prevention), previous cardiovascular events, cardiovascular events after statin termination, time for termination of statin treatment, name of the physician terminating the statin treatment, and reason for termination (if stated). 

### 4.3. Main Outcome

The main outcome was whether there was a significant difference between men and women in the time-point of statin termination in relation to time of death.

### 4.4. Statistical Analysis

As this is a retrospective observational study, no power calculations was performed. Statistical analyses were performed using Graph Pad Prism vs 8.0 (GraphPad Software, San Diego, USA) and for the attempt with the multivariate analysis SPSS was used. Comparisons of time-point of termination of statin treatment between men and women was performed using Mann–Whitney test. Mann–Whitney test was also used for comparison of all continuous variables and Fisher’s exact test for binary variables. 

### 4.5. Ethical Statement

The retrospective review of medical record was approved by the Regional Ethical Committee 2018/1798-31. Written informed consent not possible to collect since all patients were deceased. 

## 5. Conclusions

In conclusion, this study show that statin treatment was discontinued earlier in women than in men with advanced cancer and the time-point for deprescribing was not affected by age or indication for statin use. Notably, there was no difference in cardiovascular events between men and women after statin discontinuation.

## Figures and Tables

**Table 1:** Demographic data of the 149 statin users in the study cohort. *p*-Value shows statistical difference between men and women. Statistical analysis was performed with Fischers exact test for binary outcome and Mann–Whitney-U test for continuous data. *p* < 0.05 is considered as statistically significant. IQR = interquartile range, ns = not significant.

Patients Characteristics	Men (*n* = 107)	Women (*n* = 42)	*p*-Value
Age (years), median (IQR)	75 (69–80)	76 (70–81)	ns
History of stroke, *n* (%)	19 (18%)	4 (10%)	ns
History of myocardial infarction, *n* (%)	38 (36%)	7 (17%)	<0.05
Indication for statin use			
Primary prevention, *n* (%)	51 (48%)	31 (74%)	<0.01
Secondary prevention, *n* (%)	56 (52%)	11 (26%)	<0.01
Type of statin	Men (*n* = 107)	Women (*n* = 42)	
Simvastatin, *n* (%) Median dose	77 (72%) 20 mg	31 (74%) 20 mg	ns
Atorvastatin, *n* (%) Median dose	29 (27%) 40 mg	11 (26%) 30 mg	ns
Rosuvastatin, *n* (%) Median dose	1 (1%) 10 mg	0	
Type of cancer, *n*	Men (*n* = 107)	Women (*n* = 42)	
Lung	22	12	ns
Gastrointestinal	16	4	ns
Pancreas, liver, gallbladder	12	5	ns
Breast	0	6	NA
Urological	12	0	<0.05
Gynaecological	NA	8	NA
Prostate	21	NA	NA
Hematological	5	4	ns
Head–Neck	4	1	ns
Brain tumour	4	0	ns
Esophageal	5	0	ns
Melanoma	3	2	ns
Other	3	0	ns

**Table 2:** Time-point for statin-termination and outcome in cardiovascular events after statin termination. Comparison between men and women and if the indication for statin treatment was primary prevention or secondary prevention, i.e., treatment initiated after previous cardiovascular event. Statistical analysis were performed by Mann–Whitney-U test. MI = myocardial infarction, IQR = interquartile range.

Indicaton for Statin, Primary/Secondary Prevention:	Primary	Secondary	*p*-Value
Men (*n* = 107)	Men (*n* = 51)	Men (*n* = 56)	
Months before death, median (IQR)	1.5 (0.5–4.0)	1.0 (0.5–3.75)	0.43
Women (*n* = 42)	Women (*n* = 31)	Women (*n* = 11)	
Months before death, median (IQR)	3.5 (0.5–7.0)	2.5(1.5–11)	0.60
Primary prevention group	Men (*n* = 51)	Women (*n* = 31)	
Months before death, median (IQR)	1.5 (0.5–4.0)	3.5 (0.5–7.0)	0.14
Secondary prevention group	Men (*n* = 56)	Women (*n* = 11)	
Months before death, median (IQR)	1.0 (0.5–3.75)	2.5 (1.5–11)	0.06
	Men (*n* = 107)	Women (*n* = 42)	
Cardiovascular events after statin termination	1 (1%) MI	1 (2%) Stroke	ns
Cancer as cause of death	97 (91%)	40 (95%)	ns

## Data Availability

Raw data is available from the corresponding author upon request.
